# Shear Stress Induces Differentiation of Endothelial Lineage Cells to Protect Neonatal Brain from Hypoxic-Ischemic Injury through NRP1 and VEGFR2 Signaling

**DOI:** 10.1155/2015/862485

**Published:** 2015-10-05

**Authors:** Chia-Wei Huang, Chao-Ching Huang, Yuh-Ling Chen, Shih-Chen Fan, Yuan-Yu Hsueh, Chien-Jung Ho, Chia-Ching Wu

**Affiliations:** ^1^Institute of Basic Medical Sciences, National Cheng Kung University, No. 1 Daxue Road, Tainan 701, Taiwan; ^2^Institute of Clinical Medicine, National Cheng Kung University, No. 1 Daxue Road, Tainan 701, Taiwan; ^3^Department of Pediatrics, Taipei Medical University, No. 250 Wuxing Street, Taipei 110, Taiwan; ^4^Institute of Oral Medicine, National Cheng Kung University, No. 1 Daxue Road, Tainan 701, Taiwan; ^5^Department of Occupational Therapy, I-Shou University, No. 1 Syuecheng Road, Kaohsiung 824, Taiwan; ^6^Division of Plastic Surgery, National Cheng Kung University Hospital, No. 138 Sheng Li Road, Tainan 701, Taiwan; ^7^Department of Cell Biology and Anatomy, National Cheng Kung University, No. 1 Daxue Road, Tainan 701, Taiwan; ^8^Department of Biomedical Engineering, National Cheng Kung University, No. 1 Daxue Road, Tainan 701, Taiwan; ^9^International Research Center for Wound Regeneration and Repair, National Cheng Kung University, No. 1 Daxue Road, Tainan 701, Taiwan

## Abstract

Neonatal hypoxic-ischemic (HI) brain injuries disrupt the integrity of neurovascular structure and lead to lifelong neurological deficit. The devastating damage can be ameliorated by preserving the endothelial network, but the source for therapeutic cells is limited. We aim to evaluate the beneficial effect of mechanical shear stress in the differentiation of endothelial lineage cells (ELCs) from adipose-derived stem cells (ASCs) and the possible intracellular signals to protect HI injury using cell-based therapy in the neonatal rats. The ASCs expressed early endothelial markers after biochemical stimulation of endothelial growth medium. The ELCs with full endothelial characteristics were accomplished after a subsequential shear stress application for 24 hours. When comparing the therapeutic potential of ASCs and ELCs, the ELCs treatment significantly reduced the infarction area and preserved neurovascular architecture in HI injured brain. The transplanted ELCs can migrate and engraft into the brain tissue, especially in vessels, where they promoted the angiogenesis. The activation of Akt by neuropilin 1 (NRP1) and vascular endothelial growth factor receptor 2 (VEGFR2) was important for ELC migration and following *in vivo* therapeutic outcomes. Therefore, the current study demonstrated importance of mechanical factor in stem cell differentiation and showed promising protection of brain from HI injury using ELCs treatment.

## 1. Introduction

Hypoxic and ischemic (HI) brain injuries which result from lacking of oxygen or blood supply lead to permanent neuron damage and neurological deficit. During birth, HI insults in developing brain, such as asphyxia and ischemic stroke, are the leading cause of neonatal mortality and lifelong functional loss among newborns [1]. The underlying mechanism of this devastating disease is excito-oxidative cascade, including increased oxidative stress, inflammation, and cell death, which is followed by the disruption of brain neurovascular unit and further damage the tissue in ischemic penumbra [2, 3]. It suggests that neuron and vessels are interdependently related to each other [4]. Maintaining the integrity of neurovascular structure after HI brain injury is crucial for preventing brain damage and functional loss [5]. However, there is no effective therapy for treating neonatal HI brain.

Endothelial progenitor cells (EPCs) in circulation system are positively correlated with the outcome of hypoxic brain injury, and the more EPCs in circulation showed better recovery [6]. The definitions of EPCs are varied, but most of the studies agree with the classification of early EPCs and late EPCs. Both kinds of the cell express the surface antigen such as Flk, vWF, CD31, Tie2, and VE-Cadherin [7]. Upon endothelial damage, releasing of proangiogenic factors, such as vascular endothelial growth factor (VEGF), mobilizes EPCs from bone marrow and promotes its angiogenic function [8, 9]. The EPCs migrate to the hypoxic region and differentiate into mature endothelial cells (ECs) to maintain structure and function of vessel [10]. The EPCs are also capable of promoting reendothelialization, angiogenesis, and vasculogenesis and improving regeneration and function in hypoxic injured organs [10–12]. Transplantation of* ex vivo* expanded EPCs or ECs showed therapeutic effect in several disease models, including neonatal HI brain injury, stroke, myocardial infarction, and vascular injury after angioplasty [13–17]. The outcome is mainly accomplished through reendothelialization, neovascularization, and reduction of the infarction region. The recruitment and incorporation of the injected EPCs into the ischemic region are essential for the beneficial effect [18].

Although the usage of EPCs is promising in brain therapy, the shortage of autologous EPCs limits its clinical application. We previously demonstrate the induction of endothelial differentiation by synergistic biochemical and biomechanical stimulations in human placenta-derived multipotential cells (PDMCs) [19]. The application of endothelial growth medium for 3 days promotes the expression of early endothelial markers, such as VEGFR1 and VEGFR2, in PDMCs. Then, the mechanical shear stress further induces the mature EC markers and functions. Adipose-derived stem cells (ASCs), having similar characteristic with mesenchymal stem cells, are a potential source of autologous stem cell. ASCs are one of the multipotent stem cells which can be differentiated into endothelial, neural, osteogenic, chondrogenic, myogenic, and adipogenic cells under specific induction [20–22]. In current study, we are interested in whether the environmental cues, including both chemical and mechanical, can promote endothelial differentiation in human ASCs and their therapeutic potential in prevention of brain from HI injury. Although the endothelial differentiation is induced using synergistic stimulations in human PDMCs, the population of early and late EPCs is not fully separated as the circulating EPCs isolated from blood or bone marrow. We use the term of endothelial lineage cells (ELCs) to indicate the mixture endothelial population for direct cell transplantation after synergistic stimulation without sorting. Here, we reported that the neuropilin1 (NRP1) and VEGF receptor 2 (VEGFR2) signals mediated cell migration under hypoxic condition, which can reduce infarction size and preserve the neurovascular structure after HI injury in neonatal brain.

## 2. Materials and Methods

### 2.1. Isolation of Human Adipose-Derived Stem Cells

Human liposuction aspirates were obtained from healthy donors with informed consent to protect the information and rights of patients as approved in accordance with procedures of the institutional review board of the National Cheng Kung University Hospital (NCKUH). The human ASCs were isolated following the protocol described previously [20]. Briefly, the aspirates were washed by phosphate buffered saline and then were incubated with 0.075% collagenase type II (Invitrogen) at 37°C. The infranatant was mixed with Dulbecco's modified Eagle's medium (DMEM, Invitrogen) which contained 10% fetal bovine serum (FBS, HyClone) to stop the activity of collagenase, and was centrifuged for 10 min at 1000 rpm. The cells in pelleted fraction were harvested and cultured in DMEM supplemented with 10% FBS and 1% penicillin/streptomycin (Invitrogen).

### 2.2. Induction of Endothelial Differentiation and Functional Assessments

The combined chemical and mechanical stimulation was used to induce the ELCs as previously established induction protocols [19]. For the induction of early EPCs, the ASCs were seeding onto fibronectin (30 mg/mL, Sigma) coated glass slide to reach 80–90% confluence and then cultured in endothelial cell growth medium (EGM, Lonza) for 3 days under static condition. For the induction of late EPCs, the early EPCs were subjected to the laminar shear stress (LSS, 12 dyn/cm^2^) for 24 hours immediately after the EGM induction. The LSS was created by a flow chamber which allowed medium to flow through the cell-seeding glass slide and applied LSS to cells [19]. The endothelial differentiation was confirmed by the expression level of VEGFR2 (Flk) and von Willebrand Factor (vWF) using reverse transcription polymerized chain reaction (RT-PCR) according to the previous study [19]. The level of producing nitric oxide (NO) by ASC and ELC was measured by colorimetric (Nitric Oxide Assay Kit, Abcam) according to the user protocols. The human umbilical vein endothelial cells (HUVEC) were used as the positive control.

Forming tube-like structure which is an important indicator of endothelial function was measured in ASCs, early EPCs, and ELCs as previously described [19]. In brief, the cells (1 × 10^5^ cells) were trypsinized and resuspended with 1 mL complete medium and added onto the polymerized Matrigel (BD Biosciences). After incubation in 5% CO_2_ incubator for 5 hours, the formation of tube-like structure was observed and quantified in the tube length using phase contrast microscope.

### 2.3. Animal Model of Neonatal HI Brain Injury and Cell Therapy

The Sprague-Dawley (SD) rats were provided by the animal center in NCKU with the approval of the experiment procedure by the Institutional Animal Care and Use Committee at NCKU. Neonatal HI brain injury is created according to previous study [17]. Concisely, postnatal day 7 (P7) SD rat pups were anesthetized with 2.5% halothane, and the right common carotid artery was permanently ligated using 5-0 surgical silk. One hour after surgery, the pups were subjected to hypoxia (8% oxygen and 92% nitrogen) for 2 hours in an airtight chamber in which the temperature was maintained at 37°C. The rat pups were randomly assigned into 3 groups which received different treatment, including phosphate buffered saline (PBS) injection or transplantation of ASCs or ELCs. Different treatment was given by intraperitoneal administration right before and after the exposure of hypoxia, with the dosage of 1 × 10^5^ cells/100 *μ*L each time. The pups returned to dam for recovery for one week before the brain samples were harvested.

### 2.4. Brain Damage and Neurovascular Structure Measurements

Damage severity of brain was determined by the volume of ischemic region on outer surface and Nissl staining at P14. The rat brains were perfused with normal saline and 4% paraformaldehyde. Upon isolation, each brain was given a score based on the lesion size of outer surface of brain. The size of the lesion was divided into 3 grades, score one (lesion size less than 1/3 hemisphere), score two (the lesion size greater than 1/3 but lesser than 1/2), and score three (the lesion size greater than 1/2 hemisphere) [23]. After dehydration by 30% (w/v) sucrose in 0.1 M PBS and frozen tissue matrix (OCT, Leica) embedding, the brain samples were coronally sectioned into 20 *μ*m-thick slice for the following staining. The corresponding plates 15, 18, 27, 31, and 39 in rat brain according to the rat brain atlas were stained with 0.1% cresyl violet solution for 1 hour to reveal neurons [24].

The integrity of neurovascular structure was assessed by immunofluorescence staining. The brain slices were incubated with the primary antibodies against neuronal nuclear antigen (NeuN, 1 : 200, Abcam) for identifying neuron and against rat endothelial cell antigen-1 (RECA, 1 : 200, Abcam) for identifying vessels. To further verify the mechanism of the protection effect of different treatment, the marker of angiogenesis, isolectin IB4 antibody (IB4, 1 : 200, Invitrogen), was used to visualize new forming vessels. The antihuman nuclei antibody (hChromatin, 1 : 100, Millipore) was utilized to recognize transplanted human cells in rat brain. The primary antibodies were labeled by fluorescent-labeled secondary antibodies (1 : 200, Invitrogen), and the signals were observed at excitation-emission wavelengths of nm 470 to 505 nm and 596 to 615 with a tissue scanning fluorescent microscope (BX51, Olympus) using a 20x objective lens.

### 2.5. *In Vitro* Hypoxia and Boyden Chamber Migration Assay

The ability of stem cells to migrate into lesion site is important for tissue protection and regeneration. The injured brain area will release environmental cue to trigger the migration of the stem cells. We use Boyden chamber assay to compare the chemotaxis and transmigration ability of different cells. Shortly, different cells (2 × 10^4^ per well) are loaded into the upper compartment of Boyden chamber; the migration ability of the cells through 8 *μ*m-pore membranes (Neuro Probe) to the lower compartment which filled with medium with or without hypoxia mimetic reagent desferrioxamine (DFO, 50 mM, Sigma) will be measured. The cells are cultured in Boyden chamber for 6 hours, and then the cells on the lower surface of membrane will be fixed by 4% paraformaldehyde (Sigma) and stained with Giemsa to quantify the transmigrated cell numbers. To further clarify the mechanism of boosting migration ability of ELCs under hypoxic condition, the ELCs were pretreated with neuropilin-1 (NRP1) blocking peptide (DG2, 20 *μ*M, Digitalgene) or inhibitor of VEGFR (BIBF1120, 50 nM) 1 hour before being loaded into the chamber. The migrating cell area was quantified by ImageJ software.

To further illustrate the underlying signaling under hypoxic condition, the* in vitro* hypoxic condition was achieved by treating the ASCs or ELCs with 50 mM DFO. The cells were cultured in hypoxic condition for 6 hours with or without the pretreatment of DG2 (20 *μ*M) for 1 hour, followed by the collection of cell lysates. The gene expression level of VEGFR1, VEGFR2, CD31, vWF, angiopoietin 1 (ANG1), and neuropilin 1 (NRP1) was measured using RT-PCR with the primer sequence listed in previous study [19]. The activation of Akt and ERK pathway was analyzed by western blotting following the steps described before using the primary antibody against phospho-Akt (p-Akt, 1 : 500, Cell Signaling), Akt1 (1 : 100, Santa Cruz), phosphor-ERK (p-ERK, 1 : 2000, Cell Signaling), ERK2 (1 : 200, Santa Cruz), and *β*-actin (1 : 10000, Sigma) [25]. The p-Akt (1 : 50) and p-VEGFR2 (1 : 50) antibodies were also used in immunofluorescent staining to demonstrate the phosphorylation levels for regenerative tissue with the treatment of ASC or ELC after HI injury.

### 2.6. Statistical Analyses

For all experiments at least 3 independent groups were performed to demonstrate the consistency outcome. All data were expressed as the mean ± standard SEM. Statistical analysis was performed using the one-way analysis of variance (ANOVA) with Fisher's test of the Origin statistic software (version 8.5, OriginLab). The statistical significance was defined as *p* < 0.05.

## 3. Results

### 3.1. Inducing Endothelial Differentiation from ASCs

**Figure 1:**
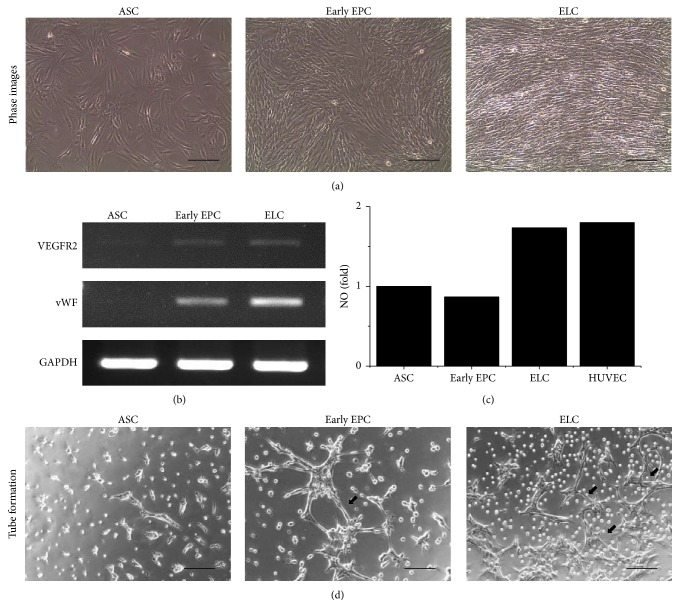
The upregulation of endothelial gene expressions and functional characteristics in different endothelial induction phases. After treating adipose-derived stem cells (ASCs) with endothelial growth medium, the early endothelial progenitor cells (early EPCs) remain spindle-shape morphology, while the subsequential application of laminar shear stress (LSS) induced the endothelial lineage cells (ELCs) to align parallel to the flow direction (a). The early EPCs showed increase of VEGFR2 expression and slight vWF induction (b). Application of LSS further induced the vWF expression in ELCs. The ELCs but not early EPCs were capable of producing NO (c). The ELCs showed better ability to form tube-like structure than undifferentiated ASCs and early EPCs (d). *N* = 5, Scale bar: 200 *μ*m.

The endothelial differentiation for early EPCs and ELCs was induced from human ASCs using biochemical and biomechanical stimulations. After EGM induction, the early EPCs did not show obvious change in morphology and remained spindle shaped which was similar to undifferentiated ASCs (Figure 1(a)). Instead, with the addition of mechanical shear stress after EGM induction, the ELCs sensed the direction of LSS and undergo morphological change that paralleled to the flow direction (Figure 1(a)). The gene expression level of endothelial lineage markers, including VEGFR2 and vWF, was increased in both early EPCs and ELCs with slightly higher expression in ELCs (Figure 1(b)). The production of nitric oxide in different induced cells was assessed by commercial NO kits with positive reference of human umbilical vein endothelial cells (HUVEC). After the stimulation of shear stress, the ELCs were capable of producing NO with the level similar to HUVEC (Figure 1(c)). To further confirm the function of the differentiated cells, we performed tube formation assay. Undifferentiated ASCs hardly form tube-like structure after being cultured on Matrigel for 5 hours. Between early EPCs and ELCs, the ELCs showed better capacity in interconnecting with each other (as indicated by arrows, Figure 1(d)), which indicated the greater angiogenic potential of ELCs more than early EPCs. The result revealed the beneficial effect of mechanical shear stress on ASCs differentiation into endothelial lineage. The application of shear stress improved EC functions of ELCs. Therefore, we used ELCs for the following studies.

### 3.2. ELCs Transplantation Protects Brain against HI Injury

**Figure 2:**
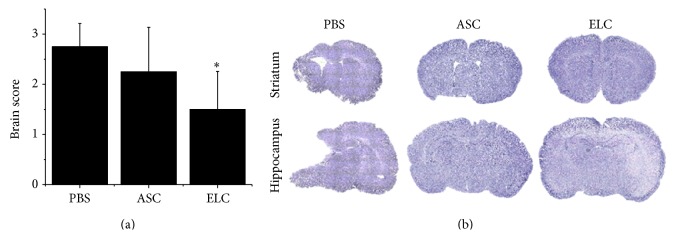
The ASCs and ELCs were transplanted into the neonatal rats to investigate the protection of cell-based therapy in hypoxic-ischemic (HI) brain injury. Significant preservation of brain damage was observed in ELCs treatment as compared to the PBS injection (a). Both ASCs and ELCs therapies showed prevention of brain loss in Nissl staining after HI injury (b). *N* = 6, ^*∗*^ *p* < 0.05 compared to PBS-treated rats.

The therapeutic effect of ASCs and ELCs was tested using neonatal HI brain injury model in rats. The severity of brain injury was compared among the rat pups receiving different treatments, including PBS, ASCs, and ELCs. The brain score determined based on infarction size on the outer surface of brain showed sever damage after HI insult in PBS group (Figure 2(a)). Transplantation of ASCs slightly reduced the brain score, while the ELCs significantly decreased the infarction size as compared to the result of injecting PBS (Figure 2(a)). Similar outcome in gross structure was observed by Nissl staining which visualized the alive neurons. Obvious brain loss after HI injury was preserved by ASCs or ELCs transplantation (Figure 2(b)).

**Figure 3:**
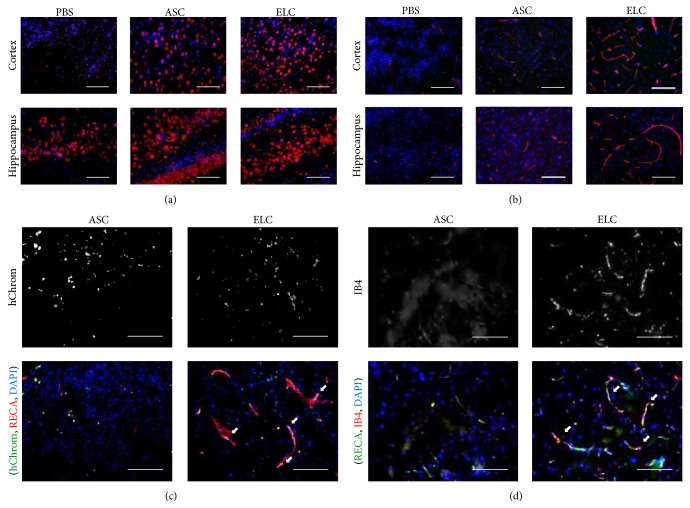
The immunofluorescence staining revealed the improvement of neurovascular structure with ELCs treatments in HI injured brain. The number of neurons was increased in cortex area with both ASCs and ELCs treatments using NeuN staining (a). The significant preservation of vascular structure was observed in both cortex and hippocampus by RECA staining in ELCs transplantation (b). The staining of hChromatin (hChrom) indicated the homing of transplanted cells into the damaged brain (c). The triple staining of hChrom (green) with vessels (RECA, red) and all cell nuclei (DAPI, blue) indicated the engraftment of transplanted ELCs to the vascular structure. The angiogenesis was assessed by isolectin B4 (IB4) staining and showed promising enhancement in ELCs treatments (d). The colocalization of IB4 (red) with RECA (green) indicated the promotion of angiogenesis after application of ELCs. *N* = 4, Scale bar: 100 *μ*m.

We further investigated the integrity of neurovascular structure by immunofluorescence staining of specific antibodies against neuron (NeuN) (Figure 3(a)) and vessel (RECA) (Figure 3(b)). Significant decrease of neuron number in both primary cortex and hippocampus was detected in PBS injected animals after HI injury (Figure 3(a)). Similarly, the density and length of vessel were also reduced in HI-brain without cell therapy (Figure 3(b)). Cell transplantation showed preservative effect on the neurovascular structure in both brain areas (Figures 3(a) and 3(b)). Interestingly, though the gross structure was preserved by ASCs transplantation, the ASCs had lesser protective effect to the neurovascular structure as compared to the effect of ELCs. The migration and engraftment of transplanted cells was confirmed using anti-hChromatin antibody (hChrom) to visualize the location of human ASCs or their derived ELCs. In the rat pups receiving cell therapy, the hChrom positive cells were found in the peri-infarction area of injured hemisphere (as indicated by arrows, Figure 3(c)). The number of engrafted cells was similar in the brains with transplantation of either ASCs or ELCs. This indicates both ASCs and ELCs can be home to the damage brain tissue after HI injury. However, the ELCs were the major contributor to restore vascular structure as showed by the triple staining of engrafted cells (hChrom, green), vessel (RECA, red), and all cell nuclei (DAPI, blue). To further address the contribution of angiogenesis in HI-injured brain, the immunofluorescence staining using angiogenesis marker (IB4) was evaluated in the brain received ASCs or ELCs transplantations (Figure 3(d)). There was almost no IB4 positive vessel in the ASCs treated brains, suggesting that ASCs may not contribute to the formation of new vessels. Instead, the IB4 expression (red) was prominently observed in the ELCs treated brain highly colocalized to the vessels (RECA, green), which indicated the promotion of neovascularization by ELCs (as indicated by arrows, Figure 3(d)). The results in vascular protection in brain injury explained in part that the transplantation of ELCs has better therapeutic effect than ASCs.

### 3.3. Promoting Migration Ability of ELCs under Hypoxic Condition

**Figure 4:**
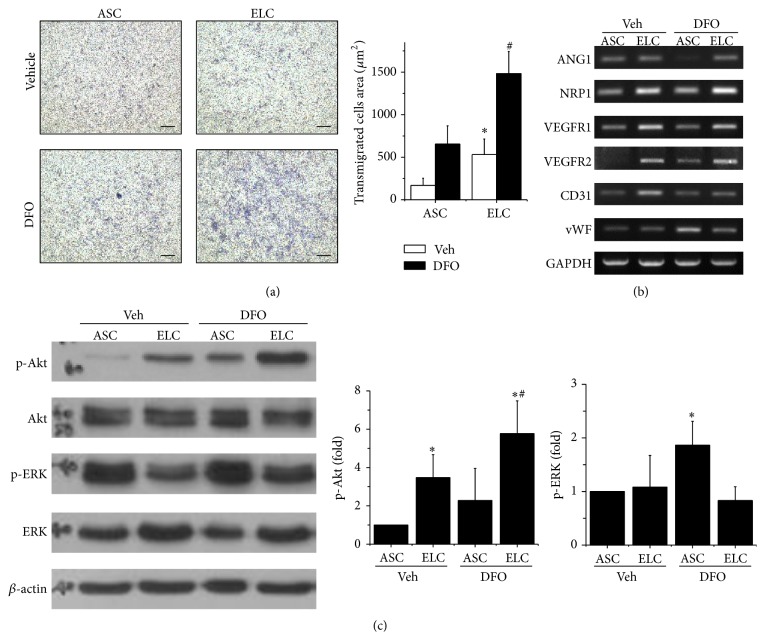
Hypoxia promoted the migration ability of cells, especially in ELCs. The transmigration of ASCs and ELCs was measured by* in vitro* Boyden chamber assay with or without the application of hypoxia mimetic reagent (DFO). The cell mobility was significantly increased in ELCs under the hypoxia (a). The NRP1 and VEGFR2 were increased in ELCs and further enhanced with DFO treatment (b). The phosphorylation of Akt (p-Akt) was increased in ELCs and also enhanced under hypoxia (c). *N* = 3, Scale bar: 200 *μ*m. ^*∗*^ *p* < 0.05 compared to undifferentiated ASCs under normoxia. ^#^ *p* < 0.05 compared to undifferentiated ASCs under hypoxia.

The HI injured brain created an* in vivo* local hypoxic environment that may have different responses in ASCs and ELCs. We further used* in vitro* Boyden chamber assay to investigate themigration ability of various cells under normal and hypoxic conditions. The transmigrated cells were labeled in dark blue color after migrating from the loaded upper compartment of chamber to the lower compartment with or without adding DFO for studying the cell mobility in normoxia (vehicle) and hypoxia (DFO) (Figure 4(a)). The ELCs showed significantly higher cell mobility than ASCs in regular culture condition (normoxia). The mobility of ELCs but not ASCs was remarkably boosted under hypoxia (Figure 4(a)). To illustrate the mechanism of this beneficial effect in ELCs, the gene expressions related to angiogenesis and migration were measured by RT-PCR for ANG1, NRP1, VEGFR1, VEGFR2, CD31, and vWF under normoxia or under hypoxia in DFO treatment (Figure 4(b)). The changes of expression patterns in NRP1 and VEGFR2 indicated the involvement of NRP1 and VEGFR2 signals in ELCs that may response to the hypoxia. The downstream signaling pathways of NRP1 and VEGFR2 were evaluated by the phosphorylation levels of Akt and ERK using western blot (Figure 4(c)). The level of phosphorylated Akt (p-Akt) was significantly increased in ELCs as compared to the level in ASCs under normoxia, and it was further promoted under hypoxia in both ASCs and ELCs. In contrast, ERK was highly phosphorylated in ASCs under both normoxia and hypoxia but decreased the p-ERK levels after being differentiated into ELCs after the synergistic treatment of EGM and LSS (Figure 4(c)). The result suggested the NRP1 and VEGFR2 may participate in promoting the transmigration under hypoxia through phosphorylation of Akt pathway.

### 3.4. Interplays of NRP1 and VEGFR2 Signaling in Migration and Differentiation

**Figure 5:**
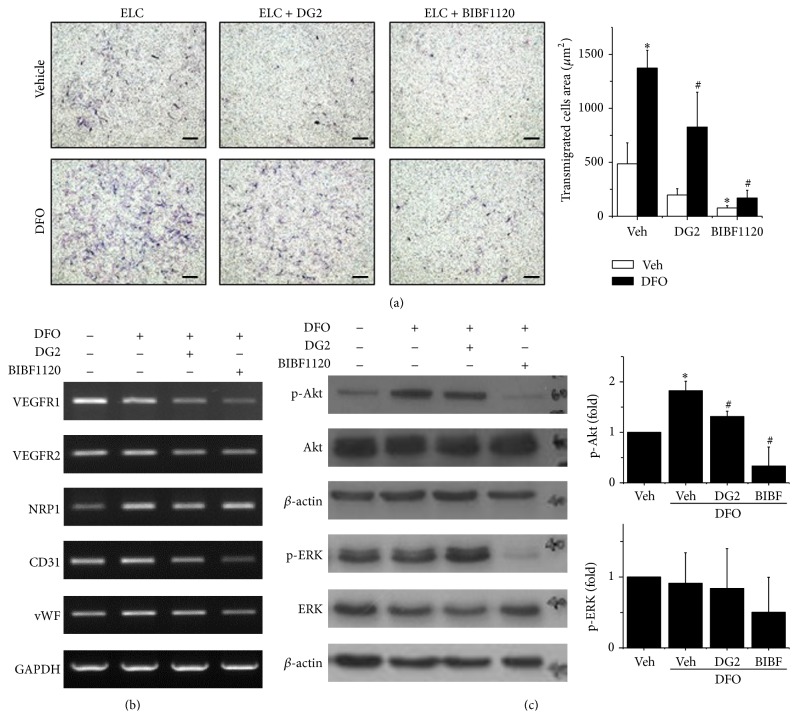
The enhancement of cell migration under hypoxia in ELCs was inhibited by using specific inhibitors to block the NRP1 (DG2) and VEGFR (BIBF1120) signaling (a). Blockage of VEGFR by BIBF1120 decreased the endothelial gene expressions (b). When treating the hypoxic ELCs with NRP1 inhibitor (DG2), both the p-Akt and total Akt were decreased (c). The treatment of VEGF inhibitors totally abolished the Akt and ERK signals. *N* = 3, Scale bar: 200 *μ*m. ^*∗*^ *p* < 0.05 compared to ELCs under normoxia. ^#^ *p* < 0.05 compared to ELC under hypoxia.

To confirm the role of NRP1 and VEGFR2 in promoting ELCs migration and remaining of endothelial differentiation under hypoxic condition, blockage of NRP1 or VEGFR signals in ELCs was tested by using specific NRP1 blocking peptides (DG2) or a small molecular targeting tyrosine kinase receptor (BIBF1120), respectively. Under regular condition (normoxia), the transmigration ability of ELCs was slightly decreased with the inhibition of NRP1 (DG2) or VEGFR (BIBF1120) signaling (Figure 5(a)). The enhancement of ELCs migration by hypoxia was inhibited by DG2 treatment and totally abolished as treated with BIBF1120. The beneficial effects of hypoxia in endothelial gene expressions for VEGFR1, VEGFR2, NRP1, CD31, and vWF were slightly altered by DG2 treatments (Figure 5(b)). Moreover, the inhibition of VEGFR signaling via BIBF1120 treatments significantly reduces the gene expressions of endothelial marker which might lead to diminish the endothelial differentiation in ELCs. The NRP1 gene expression did not alter too much when treating with DG2 or BIBF1120. These results suggested that the specific inhibitors targeted mainly the intracellualr signaling by blocking the posttranslational modification. The increase of p-Akt in ELCs under hypoxia was inhibited in DG2 treatment and was totally abolished in BIBF1120 treatments (Figure 5(c)). It is noticed that not only the p-Akt but also the total form of Akt (Akt) was decreased when applying NRP1 or VEGFR2 inhibitors to the ELCs. The decrease of p-ERK in ELC under hypoxia was restored with DG2 treatment but was further decreased with BIBF1120 treatment (Figure 4(c)). These results demonstrated involvement of both NRP1 and VEGFR2 in the beneficial effects of ELCs, but the interplays of transmigration and endothelial differentiation are complicated. The NRP1 majorly contributed to the migration ability of ELCs under hypoxic condition via Akt phosphorylation. However, the VEGFR2 has important roles in ELCs for both cell migration and endothelial differentiation.

**Figure 6:**
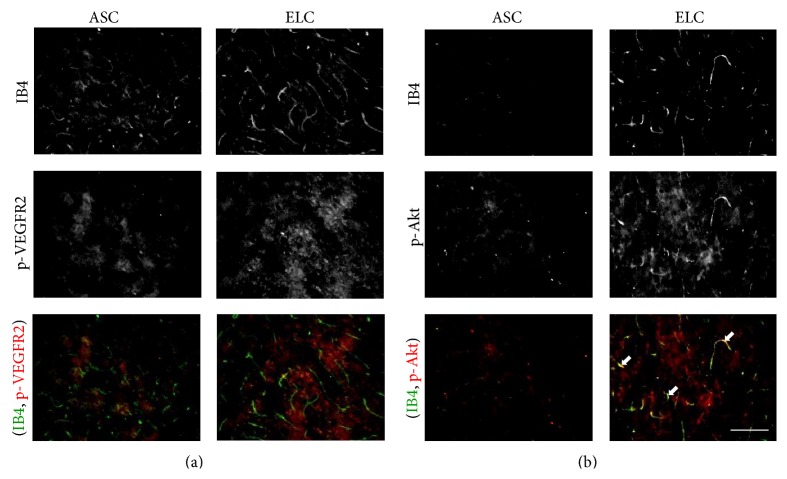
The immunofluorescence staining revealed the phosphorylation of VEGFR2 (a) and Akt (b) after application of ELCs in HI injured brain. The colocalization of p-Akt (red) with angiogenesis marker IB4 (green) indicated the involvement of Akt signaling in vascular regeneration. *N* = 4, Scale bar: 100 *μ*m.

To further confirm the signaling in brain tissue after receiving different therapeutic cells, the p-VEGFR2 and p-Akt were coimmunofluorescent staining with the angiogenesis marker IB4 (Figure 6). The application of ELCs increased both p-VEGFR2 and p-Akt in brain tissue. However, the p-VEGFR2 expression was not located on the vascular network during coimmunofluorescent staining with IB4 (Figure 6(a)). This might due to the nonspecific antibody for immunofluorescent staining. On the other hand, we found the p-Akt is partially phosphorylated to the angiogenic vessels with double positive staining of p-Akt and IB4 (Figure 6(b)). This suggests the p-Akt is involved in the regeneration of vascular network by treating with ELCs after HI injury.

## 4. Discussion

In this study, we demonstrated the ASCs can be differentiated into endothelial lineage by synergistic stimulation of growth factor and shear stress. The therapeutic effects of both ASCs and ELCs were tested in HI injured brain. Main finding of present study supported that transplantation of ELCs showed better capacity than ASCs in preserving neurovascular structure after HI brain injury in rats. The ELCs were able to migrate and incorporate into the vascular structure in brain tissue. In addition, the ELCs can promote the angiogenesis in the impaired area as compared to ASCs. The involvement NRP1 and VEGFR2 is important for ELCs to facilitate the migration ability under hypoxic condition through Akt pathway.

The angiogenic potential in early EPCs and late EPCs is comparable; there is divergence in their functions [26]. Early EPCs secrete plentiful cytokines, including VEGF, interleukin 8, hepatocyte growth factor, and granulocyte-colony stimulating factor [26, 27], while late ELCs have a better ability in survival, proliferation, and incorporation [26]. It suggests that EPCs in distinct differentiation phase may have their unique role in protecting vascular structure. The interaction between these two types of cells through autocrine and paracrine augment the effect of neovascularization in ischemic tissue [7]. The ELCs used in present study are heterogeneous containing a mix population of endothelial lineage cells, including both early EPCs and late EPCs. The synergism of the cells may be critical in the beneficial effect of ELCs therapy.

The cell-based therapy using EPCs shows protective effect on restoring blood flow and preventing tissue damage. Upon transplantation, the EPCs home the ischemic site and incorporate into the damaged vessel structure. Studies address the fact that there are two main mechanisms for the therapeutic effect of EPCs therapy. First, the EPCs have potential to differentiate into mature ECs [28]. After adhering to the injured endothelium, EPCs proliferate and directly incorporate into the endothelium to maintain the vessel structure and function [29]. Second, the engrafted EPCs secrete paracrine factor that supports the endogenous angiogenesis process, including increasing proliferation, motility, and viability of mature ECs [30]. It partially explains the reason why stem cell therapy can effectively promote regeneration while only few cells are transplanted into the tissue. In current study, we labeled the transplanted human cells in rat brain and found the successful engraftment of both ASCs and ELCs with more ELCs engrafted into the vessel structure. However, the number of the engrafted cells was not sufficient enough to replace all damaged ECs solely by ELCs. In agreement with previous studies, our study discovered that transplantated ELCs also initiated the IB4 positive vessels for triggering the angiogenesis of endogenous ECs through secreting trophic factors.

The crosstalk between ECs and neuron are prominent for the normal structure and function of nervous system. The ECs in central nerve system are highly specialized which connect tightly with each other and form blood barrier [31]. The blood-brain barrier (BBB) also maintains the function of neurons. Once the BBB disrupts result from brain injury, the increased permeability into the brain may cause neurotoxicity and harm neuronal functions [3]. Besides functioning as a barrier, ECs also actively interact with neural cells and promote neurogenesis. During the development of brain, vessels are attracted by the growth factor released by ventricular zone and participate in the formation of stem cell niche [32]. The vascular structure not only nourishes the neural stem cell but also secretes factor that increases proliferation and self-renewal of neural stem cells in stem cell niche [33]. The ECs have the capacity of secret trophic factor such as VEGF or brain-derived neurotrophic factor to promote proliferation, neural differentiation, recruitment, and viability of neurons [34, 35]. It explains the reason why restoring the integrity of vascular structure can decrease infarction and preserve neuron number after HI brain injury. In current study, we showed that the transplantation of ELCs protects the vessel from HI insult and therefore avoids compromise to the neuron. The further study can be done to verify if ELCs therapy can actually prevent damage of BBB and the paracrine effect on neurogenesis.

Several chemoattractants will be secreted in the injured tissue after HI insult to recruit circulating cells into the damaged area. VEGF is an important chemotactic factor for EPCs to participate in cell mobilization [8]. VEGF acts on EPC chiefly through binding to its receptors, including VEGFR1, VEGFR2, and NRP1 [36]. The activation of VEGF signaling not only mobilizes EPCs or ECs, but also promotes angiogenesis of the cells. Upon binding to its receptor, VEGF activates the focal adhesion kinase, PI3 kinase/Akt pathway, and MAPK pathway [37–39]. Both of NRP1 and VEGFR were the potential upstream signals for Akt activation. In lung cancer cells, the application of NRP1 specific inhibitory peptide (DG2) decreased the p-VEGFR2 expression levels [40]. It is suggested that the VEGF (VEGF165) can bind to NRP1 and then phosphorylate the VEGFR2 to trigger the NRP1/VEGFR2/PI3K/Akt signaling pathways for tumor angiogenesis and invasion. The BIBF1120 is a tyrosine kinase inhibitor. The VEGF can induce the phosphorylation of Akt at T308 and S473 in many cells including head and neck squamous cell carcinoma [41]. Therefore, the use of BIBF1120 can block the signaling through VEGFR and affect the Akt activation. In the present study, we identified the VEGFR and its coreceptor NPR1 also plays important roles in regulating ELCs migration to the HI injured brain.

## 5. Conclusion

The current study demonstrates the importance of chemical and mechanical factors in inducing the differentiation of ELCs from ASCs and the potential of using ELCs in protecting rat neonatal HI brain injury. Both ASCs and ELCs therapies can prevent the brain from HI insult by decreasing the infarction volume. Yet, ELCs showed a promising outcome in protecting the architecture and integrity of neurovascular unit. The transplantation of ELCs preserved not only the networks of vascular structure, but also the number of neurons. The mechanism for protective effect in ELCs may act through the increase of migration capacity and neovascularization after HI injury. The increase of NRP1 and VEGFR2 was identified with phosphorylation of Akt signaling in ELCs under hypoxic condition. Inhibition of NRP1 signal diminished the promotion of ELCs transmigration and Akt phosphorylation under hypoxia. Inhibition of VEGFR2 totally abolished the cell migration and endothelial differentiation in ELCs. Taken together, these results indicate that shear stress induced ELCs can be a promising cell resource for autologous cell therapy in HI brain injury.
